# The role of voltage-gated sodium channel genotypes in pyrethroid resistance in *Aedes aegypti* in Taiwan

**DOI:** 10.1371/journal.pntd.0010780

**Published:** 2022-09-22

**Authors:** Han-Hsuan Chung, Cheng-Hui Tsai, Hwa-Jen Teng, Kun-Hsien Tsai

**Affiliations:** 1 Center for Diagnostics and Vaccine Development, Centers for Disease Control, Ministry of Health and Welfare, Taipei, Taiwan; 2 Institute of Environmental and Occupational Health Sciences, College of Public Health, National Taiwan University, Taipei, Taiwan; 3 Department of Public Health, College of Public Health, National Taiwan University, Taipei, Taiwan; Liverpool School of Tropical Medicine, UNITED KINGDOM

## Abstract

**Background:**

*Aedes aegypti* is the major vector of dengue that threatens public health in tropical and subtropical regions. Pyrethroid-based control strategies effectively control this vector, but the repeated usage of the same insecticides leads to resistance and hampers control efforts. Therefore, efficient and prompt monitoring of insecticide resistance in local mosquito populations is critical for dengue control.

**Methodology/Principal finding:**

We collected *Ae*. *aegypti* in southern Taiwan in March and October 2016. We analyzed the voltage-gated sodium channel (*vgsc*) genotypes of parentals (G0) and G1 adults after cypermethrin insecticide bioassay. Our results showed that four VGSC mutations (S989P, V1016G, F1534C, and D1763Y) associated with resistance were commonly detected in field-collected *Ae*. *aegypti*. The frequencies of these four mutations in the local mosquito population were significantly higher in October (0.29, 0.4, 0.27 and 0.11) than in March (0.09, 0.16, 0.18 and 0.03). Specific *vgsc* combined genotypes composed of the one to four such mutations (SGFY/SGFY, SVCD/SVCD, SGFY/PGFD, SVCD/SGFY, PGFD/PGFD, and SVCD/PGFD) shifted towards higher frequencies in October, implying their resistance role. In addition, the cypermethrin exposure bioassay data supported the field observations. Moreover, our study observed an association between the resistance level and the proportion of resistance genotypes in the population.

**Conclusions/Significance:**

This is the first study to demonstrate the role of four-locus *vgsc* genotypes in resistance evaluation in a local *Ae*. *aegypti* population in Taiwan. This alternative method using resistance-associated genotypes as an indicator of practically insecticide resistance monitoring is a useful tool for providing precise and real-time information for decision makers.

## Author summary

Dengue outbreaks occur annually in Taiwan, and pyrethroid insecticides are commonly used to reduce mosquito density. Insecticide resistance of mosquitoes is commonly observed in the field and threatens vector control programs. Here, we analyzed the association between the combined *vgsc* genotype and resistance phenotype based on field surveillance data in March and October and a cypermethrin exposure bioassay. Resistance-attributable specific *vgsc* genotypes were proposed. Using the combined *vgsc* genotype rather than each *vgsc* allele is recommended for better resistance prediction to provide real-time information for control program managers.

## Introduction

*Aedes aegypti* is the major vector of dengue virus, zika virus, chikungunya virus and yellow fever virus. Among them, dengue virus causes dengue fever and severe dengue with high mortality when medical resources are lacking in tropical and subtropical regions. Approximately 3.9 billion of people living in more than 120 countries are at risk of dengue virus infection. Each year, about 390 million people suffer from dengue infection, which causes health and economic burdens around the world [1,2]. In Taiwan, *Ae*. *aegypti* is limitedly distributed in southern Taiwan, especially in Tainan city, Kaohsiung city, Pingtung County [3]. According to the National Disease Notification System, dengue has occurred annually in Taiwan in the past two decades. Moreover, record levels of indigenous dengue cases were reached in 2015, with 43,419 cases, and more than 98.5% of the cases occurred in *Ae*. *aegypti* distributed areas [3]. However, dengue is not considered an endemic disease in Taiwan because most dengue outbreaks emerge from imported dengue cases in late spring or early summer; subsequently, viruses are introduced into a local vector population from infected visitors and local returning residents [4]. Dengue outbreaks usually end in the winter when the weather becomes cold and mosquito activities, including blood-feeding and oviposition, are extremely low [5–7]. However, overwintering of dengue outbreaks occurred in 1987–1988 (DENV-1), 2001–2002 (DENV-2), 2009–2010 (DENV-3), and 2014–2015 (DENV-1) [8,9].

To date, no specific antiviral drug is available for dengue. Although one dengue vaccine (Dengvaxia) has been approved in some endemic countries, the protection efficacy is not sufficient and is used with limitations, including the age of the recipient and serostatus to dengue virus [10,11]. This vaccine is not approved in Taiwan due to elderly dengue patients and the multiserotypes of dengue virus in circulation [12,13]. Therefore, mosquito control is still a primary strategy to combat dengue infection. Among all mosquito control measures, insecticide spraying is necessary as a rapid intervention to reduce mosquito density and interrupt transmission by killing infectious female mosquitoes. When a suspected case of dengue is reported, an insecticide space spray is carried out in the residence and working place. The predominant insecticides used are pyrethroids, including permethrin and cypermethrin. [14,15]. Pyrethroid is a synthetic analog of pyrethrum derived from *chrysanthemum* species with the characteristics of high and rapid toxicity toward target insects and relatively low adverse effects to mammals [15,16]. The killing mechanism of pyrethroid is binding to its receptor voltage-gated sodium channel (VGSC) in the nervous system of insects and causing sustained nerve activation leading to paralysis and death [17]. However, repeated exposure of mosquitoes to the same insecticide results in resistance and hampers the effort of vector control programs and should be carefully monitored to adjust the adopted strategy [18]. In Taiwan, mosquitoes resistant to different classes of insecticides with different modes have been observed in adults and larvae of *Ae*. *aegypti* in field populations [19,20].

One of the most important resistance mechanisms of pyrethroid is knockdown resistance (*kdr*). Changing single or multiple amino acids of VGSCs reduces the binding affinity of pyrethroids and subsequently raise the resistance to pyrethroids in mosquitoes [21]. To date, at least 14 amino acid substitutions in 11 VGSC codons, which comprise various haplotypes in *Ae*. *aegypti*, have been identified. Among them, V410L, I1011M, V1016G and F1534C (the positions are numbered based on house fly VGSCs; GenBank accession number: AAB47604) have been functionally confirmed for resistance [22–26]. The co-occurrence of *vgsc* mutations in a resistant mosquito is also commonly detected and believed to provide higher resistance levels [27]. For example, S989P strengthens V1016G-dependent insensitivity, T1520I was potent to reduce F1534C-dependent sensitivity, and S989P+V1016G+F1534C confers extremely high resistance to each mutation [28–30]. Therefore, surveillance of VGSC mutations in mosquito populations has been conducted to characterise resistance in *Ae*. *aegypti* with lowered susceptibility [29,31]. In Taiwan, the detection of the coexistence of V1016G and D1763Y in a permethrin-resistant strain of *Ae*. *aegypti* was first described by Chang and his colleagues in 2009 [32]. Later, four VGSC mutations [S989P (TCC to CCC), V1016G (GTA to GGA), F1534C (TTC to TGC) and D1734Y (GAC to TAC)], which comprised six haplotypes, were reported in 2019 [33]. In this previous study, a haplotype harboring S989P+V1016G conferred resistance to cypermethrin and could be a reference for resistance monitoring. However, the VGSC gene comprises two individual haplotypes in one mosquito; the relationship between the four-locus *vgsc* genotype and pyrethroid resistance deserves to be addressed. Therefore, the objective of this study was to address the association between combined (S989P, V1016G, F1534C and D1763Y) *vgsc* genotypes and cypermethrin resistance by analyzing alive and dead mosquitoes in cypermethrin exposure experiments. In addition, monitoring of the *vgsc* genotype in local populations of *Ae*. *aegypti* before and after vector control intervention was also discussed.

## Materials and methods

### Mosquito collection and maintenance

**Fig 1:**
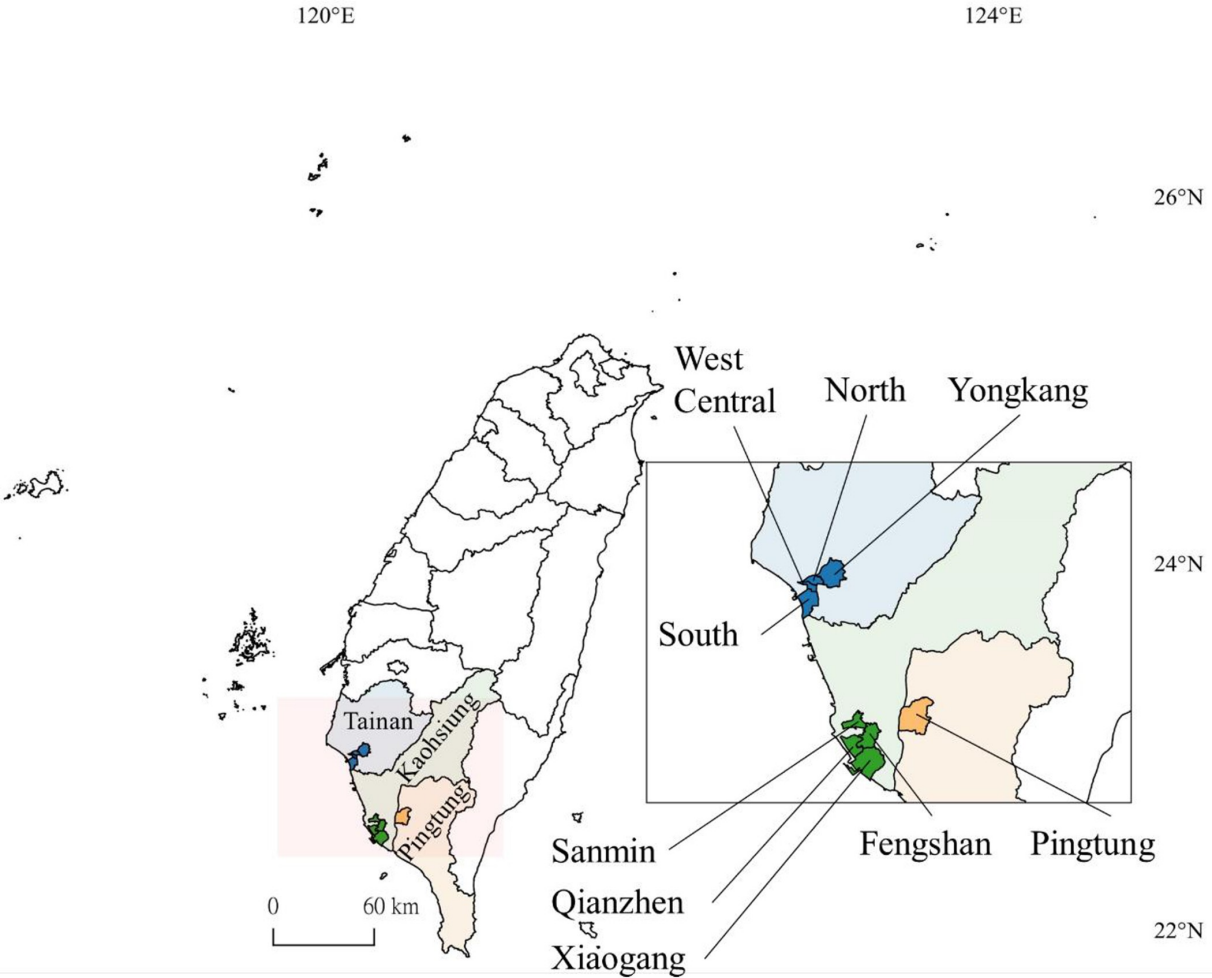
Collection sites of *Ae*. *aegypti*. Tainan City (blue color; 4 districts), Kaohsiung City (green color; 4 districts), and Pingtung County (orange color, 1 district), southern Taiwan. The map was created in QGIS 2.18.22, https://qgis.org). The base layer of the map was downloaded from Government Open Data established by National Development Council, Taiwan (https://data.gov.tw/dataset/7442).

We had an outbreak of 43,419 confirmed indigenous cases in 2015 and no outbreaks were detected after. To avoid the re-emerging of dengue in the next year, prophylactic strategies including massive chemical intervention was carried out. Therefore, it’s an opportunity to elucidate the role of *vgsc* genotypes in resistance in 2016. Immature *Aedes* mosquitoes were collected in water-filled containers in nine high dengue-risk districts of southern Taiwan, including four districts of Tainan City (West Central District, South District, North District and Yongkang District), four districts of Kaohsiung City (Sanmin District, Xiaogang District, Qianzhen District and Fengshan District), and Pingtung city of Pingtung County in March and October 2016 (Fig 1). These immature mosquitoes were brought back to the laboratory and identified for species under a dissecting microscope. Then, the mosquitoes were reared to adults (G0) or maintained to the next generation (G1) in the insectary. The detailed procedure for mosquito rearing was described in a previous study [33]. In brief, larvae were reared in a plastic pan providing a 3:1 mixture of liver powder and yeast extract daily. Adult mosquitoes were kept in an acrylic cage (30 × 30 × 30 cm; MegaView Science, Taichung, Taiwan) under conditions of a 10:14 light:dark cycle, 20–30°C and 70±10% RH. Sucrose solution (10%) was supplied. After providing blood meals, eggs were collected and hatched, and larvae were reared to adults (G1). Females of G1 were used for further insecticide exposure bioassays. G0 males and G1 females after bioassay were used for *vgsc* genotyping.

### Cypermethrin bioassay

To evaluate the cypermethrin resistance of mosquitoes, the bioassay followed the small-scale study of WHO guidelines [34]. In brief, 275 two- to five-day-old-non-blood-fed G1 female mosquitoes of each district in March and October were grouped into 25 mosquitoes per cage in a total of 11 cages, of which 10 cages of the experimental group were hung in the exposure room (~30 m^3^). The remaining cage was left in the mosquito rearing room during the experiment for the unexposed control. A susceptible lab strain Tainan1987 was tested in parallel to make sure the procedures were implemented correctly (the mortality of Lab strain>99%). Commercial cypermethrin was used owing to its common usage in vector control during dengue epidemics in southern Taiwan (Cy5 from TYENG LING Incorporation for Kaohsiung city and You-ke from JIH NONG SCIENCE CO., LTD. for Tainan city). The concentration was used according to the manufacturer’s recommendation for effective vector control (ranging from 0.01 to 0.2% w/w). Sixty microliters of diluted cypermethrin was sprayed by air atomizing nozzles (SU1A, Spraying Systems Co. U.S.). After 30 minutes of exposure, the mosquitoes were transferred into the collecting chamber (BioQuip Products Inc., Rancho Dominguez, CA, USA), supplied with 10% sucrose solution on cotton pads. The mosquitoes were subsequently moved to an incubator at 27±2°C and 75±10% RH. After 24 hours, dead or alive mosquitoes in each experiment were recorded according to the criteria described in previous WHO guidelines, and the LC_99_ of each district was calculated [35]. At the concentrations causing mortality higher than 90% in each group, we randomly collected dead mosquitoes and all that did not die. We obtained a sufficient number of alive mosquitoes only in October. To further clarify the role of *vgsc* genotype, a few samples of the lab strain of *Ae*. *aegypti* in bioassay were also included. We stored the mosquitoes at -80°C until further genotyping.

### *vgsc* genotyping

*vgsc* genotyping is based on a previous study [18]. Briefly, genomic DNA was extracted individually from field G0 males to avoid the contamination of sperm DNA in female. Because the sex determination factor and *vgsc* locus in *Ae*. *aegypti* located on the chromosome 1 and 3 separately [36,37], theoretically, the results from male DNA should not cause sexual bias. On the other hand, alive and dead G1 female mosquitoes collected in insecticide exposure bioassays were also included in *vgsc* genotyping. Genomic DNA was purified by a Qiagen QIAamp DNA purification kit (Cat. NO 51306, QIAGEN, Germany) according to the manufacturer’s instructions. Briefly, each mosquito was homogenized with a 3 mm glass bead in a 1.5 mL microcentrifuge tube for 3 min at a frequency of 30/s using a TissueLyser (Qiagen, Germany). The homogenized sample was processed, and genomic DNA was eluted with 80 μL Tris-EDTA buffer. *vgsc* was genotyped as described previously [33,38]. In brief, partial DNA fragments of domain II (S989, V1016), domain III (F1534), and domain IV (D1763) of VGSCs were amplified by 3 sets of PCR primers (S1 Table)[38] using a thermocycler (Biometra T3000, Germany). PCR was conducted with a mixture of 12.5 μL 2xPCR Master mix Solution (i-pfu) (Cat. No 25186, iNtRON BIOTECHNOLOGY, Korea), 1 μL of each forward and reverse primer (10 μM), 1 μL genomic DNA and 9.5 μL ddH_2_O in a final volume of 25 μL. PCR conditions were 94°C for 5 min, followed by 39 cycles of denaturation at 94°C for 30 s, annealing at 55°C for 30 s, extension at 72°C for 1 min, and a final extension step at 72°C for 10 min. The specific PCR amplification products were checked by electrophoresis using a 1.5% agarose gel and visualized by ethidium bromide staining. The amplicon was sent out for direct sequencing (Genomics, Taiwan) by designated sequencing primers (S1 Table) [38]. The *vgsc* genotypes of each allele [S989P (TCC to CCC), V1016G (GTA to GGA), F1534C (TTC to TGC) and D1734Y (GAC to TAC)] were visualized and confirmed using GeneStudio software (http://genestudio.com/). Based on the genotype of each allele and the six haplotypes proposed in Taiwanese *Ae*. *aegypti* in the previous study [33], the combined *vgsc* genotype was determined.

### Statistical analysis

All statistical analyses were performed with SPSS statistics version 25 (IBM, NY, USA). Unpaired Student’s *t* test was used to compare mutant allele frequencies (S989P, V1016G, F1534C, and D1763Y) between March and October and between dead and alive mosquitoes. Chi square test or Fisher’s exact test (in small sample size of some cells) was used to compare *vgsc* genotype of each allele and combined *vgsc* genotypes collected between March and October and between dead and alive mosquitoes after insecticide bioassays. The LC_99_ of cypermethrin in each group was estimated according to treated cypermethrin concentrations and the mortality corrected by Abbott’s formula. The association between the proportion of genotype and LC_99_ of cypermethrin was estimated by Pearson’s correlation model.

## Results

### *vgsc* allele and four-locus genotypes in field-collected *Aedes aegypti*

**Fig 2:**
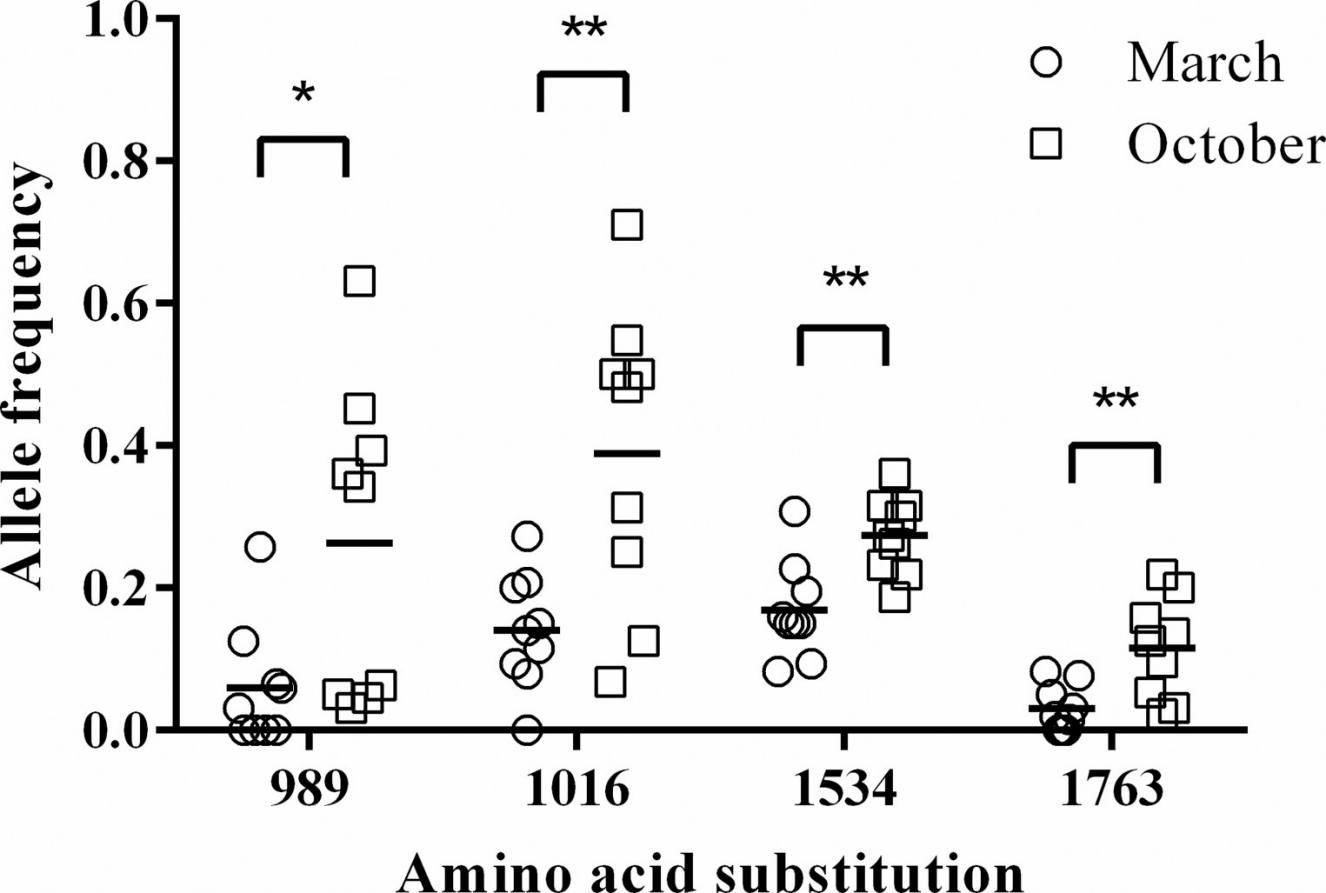
*vgsc* allele frequencies in field-collected male *Ae*. *aegypti* in March and October. All 4 mutant allele frequencies of mosquitoes collected in October were significantly (* *p* < 0.05; ** *p* < 0.01) higher than those collected in March by unpaired *t* test.

A total of 320 males (148 males in March, 172 males in October) were genotyped. Four *vgsc* allele frequencies (S989P, V1016G, F1534C, and D1763Y) were significantly higher (*p* < 0.05 or 0.01) in October (0.29, 0.4, 0.27 and 0.11) than in March (0.09, 0.16, 0.18 and 0.03) (Fig 2 and S2 Table). The mutant alleles in the 989 and 1016 (heterozygous and homozygous) were significantly higher (*p* < 0.001) in October (45, 27, 59, and 40) than in March (15, 6, 34, and 6) compared with that of the wild type (S989: 100 in October, 127 in March and V1016: 73 in October, 108 in March) (Table 1). The mutant alleles in the 1534 and 1763 (heterozygous) were slightly significantly higher (*p* < 0.05) in October than in March, while only 4 specimens with homozygote in 1763 were found. We further analyzed the genotypes composed of 4 mutations; sixteen *vgsc* genotypes were detected (Table 2). The sequence for each part of *vgsc* were deposited in NCBI GenBank (accession numbers: MK495869~ MK495876). Among them, 4 genotypes (SGFY/PGFD, SVCD/SGFY, PGFD/PGFD, and SVCD/PGFD) in local *Ae*. *aegypti* populations significantly (*p* < 0.01) increased from March to October compared with that of the wild type. 2 genotypes (SGFY/SGFY and SVCD/SVCD) slightly significantly (*p* < 0.05) increased from March to October compared with that of the wild type. Most of them had S989P or V1016G mutation except one genotype (SVCD/SVCD). The percentages of increase in these genotypes were in the ranges of 2.3 to 16.5%.

**Table 1:** The distribution of *vgsc* genotypes (for single amino acid sites) in field-collected male *Ae*. *aegypti* mosquitoes in March and in October. Underlined letter represents the mutant alleles in each position.

*vgsc* genotypes		Tainan City		Kaohsiung City		Pingtung County		Total		*p* value
		Mar.	Oct.	Mar.	Oct.	Mar.	Oct.	Mar.	Oct.	
989	S/S	54	58	51	33	22	9	127	100	-
	S/P	5	6	7	30	3	9	15	45	0.0000
	P/P	1	0	5	24	0	3	6	27	0.0001
1016	V/V	43	45	44	23	21	5	108	73	-
	V/G	16	16	14	32	4	11	34	59	0.0003
	G/G	1	3	5	32	0	5	6	40	0.0000
1534	F/F	43	40	42	48	18	8	103	96	-
	F/C	17	17	13	31	6	11	36	59	0.0260
	C/C	0	7	8	8	1	2	9	17	0.1001
1763	D/D	55	52	60	69	24	16	139	137	-
	D/Y	5	11	3	16	1	4	9	31	0.0010
	Y/Y	0	1	0	2	0	1	0	4	0.0629

**Table 2:** The distribution of combined *vgsc* genotypes in field-collected male *Ae*. *aegypti* in March and October. Genotype was described as the composition of two haplotypes and underlined letter represents the mutant alleles in each position.

No.	*vgsc* genotype (989-1016-1534-1763)	March		October		Oct.-Mar. (%)	*p* value
		no.	%	no.	%		
1	SVFD/SVFD	72	48.6	48	27.9	-20.7	-
2	SVFD/SVCD	26	17.6	8	4.7	-12.9	n.s.
3	SVFD/PGFD	10	6.8	2	1.2	-5.6	n.s.
4	SVFD/SGFY	9	6.1	4	2.3	-3.8	n.s.
5	SVCD/SGFD	6	4.1	2	1.2	-2.9	n.s.
6	SVFD/SGFD	5	3.4	2	1.2	-2.2	n.s.
7	SVFD/PVFD	1	0.7	0	0	-0.7	n.s.
8	SGFD/SGFY	0	0	1	0.6	0.6	n.s.
9	SP/VG/FC/DY*	0	0	2	1.2	1.2	n.s.
10	PGFD/PGFY	0	0	2	1.2	1.2	n.s.
11	SGFY/SGFY	0	0	4	2.3	2.3	0.0289
12	SVCD/SVCD	9	6.1	17	9.9	3.8	0.0182
13	SGFY/PGFD	0	0	8	4.7	4.7	0.0010
14	SVCD/SGFY	0	0	14	8.1	8.1	0.0000
15	PGFD/PGFD	6	4.1	25	14.5	10.5	0.0001
16	SVCD/PGFD	4	2.7	33	19.2	16.5	0.0000
Total		148	100	172	100		

* Haplotypes cannot be proposed according to previous study [33].

n.s. denotes not significant.

### *vgsc* allele and four-locus genotypes in dead or live *Ae*. *aegypti* after cypermethrin exposure bioassay

**Fig 3:**
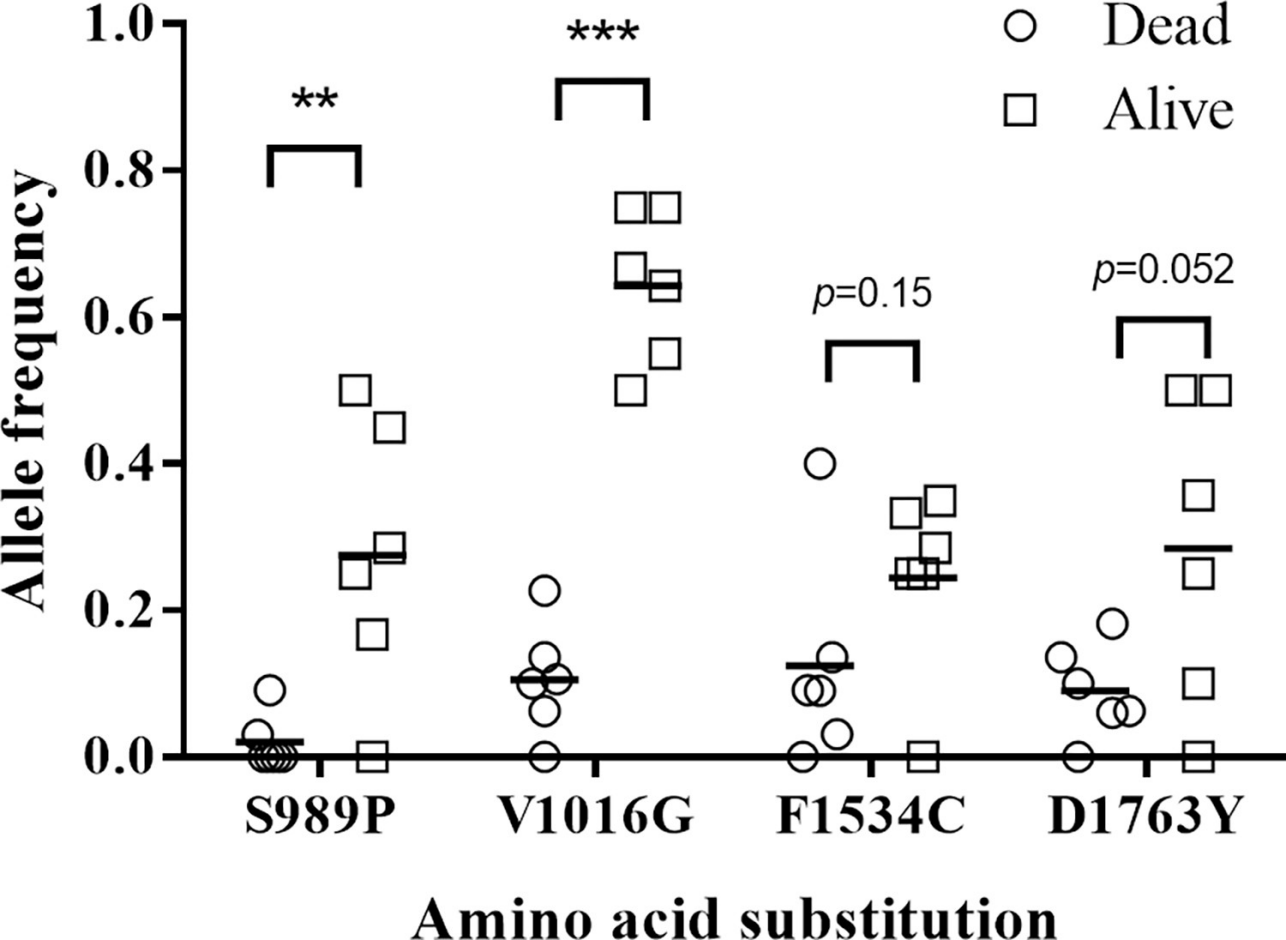
*vgsc* allele frequencies in live and dead G1 female mosquitoes after cypermethrin exposure bioassay. Two mutant allele frequencies (S989P and V1016G) in alive mosquitoes were significantly higher (**p < 0.01; ***p < 0.001) than those in dead mosquitoes by unpaired *t* test.

A total of 115 G1 females (78 dead females and 37 live females) were genotyped (S5 Table). All four *vgsc* mutation frequencies (S989P, V1016G, F1534C, and D1763Y) were higher in alive mosquitoes than in dead mosquitoes (S989P and V1016G were significantly higher) (*p* < 0.001) (Fig 3). The mutant alleles in 989 and 1016 (heterozygous and homozygous) were significantly higher (*p* < 0.01) in alive mosquitoes (22, 3, 23, and 11) than in dead mosquitoes (4, 0, 18, and 0) compared with those of the wild type (S989: 12 live, 74 dead and V1016: 3 live, 60 dead) (Table 3). The mutant alleles in 1534 heterozygous and 1763 homozygous were also significantly higher (*p* < 0.01) in alive mosquitoes than in dead mosquitoes. We further analyzed the genotypes comprising 4 mutations; 12 *vgsc* genotypes were detected (Table 4). Among them, 7 genotypes (SVCD/SVCD, SVFD/PGFD, SVCD/PGFD, SVCD/SGFY, SGFY/PGFD, SGFY/SGFY and PGFD/PGFD) of G1 mosquitoes was significantly (Fisher’s exact test, *p* < 0.01) higher in alive mosquitoes than in dead mosquitoes compared with that of the wild type. One genotype (SGFD/SVFD) was slightly significantly higher (*p* < 0.05) in alive mosquitoes than in dead mosquitoes compared with that of the wild type. Most of them had S989P or V1016G mutation except one genotype (SVCD/SVCD). The survival rates of these genotypes were in the range of 50 to 100%. When the lab strain *Ae*. *aegypti* was included in cypermethrin exposure bioassay, similar results were observed (S3 and S4 Tables).

**Table 3:** The distribution of *vgsc* genotypes (for single amino acid sites) in live and dead G1 female mosquitoes after cypermethrin exposure bioassay. Underlined letter represents the mutant alleles in each position.

*vgsc* genotypes		Tainan City		Kaohsiung City		Total		*p* value
		Dead	Live	Dead	Live	Dead	Live	
989	S/S	16	4	58	8	74	12	-
	S/P	2	5	2	17	4	22	0.0000
	P/P	0	0	0	3	0	3	0.0040
1016	V/V	12	1	48	2	60	3	-
	V/G	6	4	12	19	18	23	0.0000
	G/G	0	4	0	7	0	11	0.0000
1534	F/F	14	4	50	12	64	16	-
	F/C	2	5	10	14	12	19	0.0000
	C/C	2	0	0	2	2	2	0.1701
1763	D/D	13	4	51	21	64	25	-
	D/Y	5	3	9	5	14	8	0.1496
	Y/Y	0	2	0	2	0	4	0.0081

**Table 4:** The distribution of combined *vgsc* genotypes in live and dead G1 female after cypermethrin exposure bioassay. Genotype was described as the composition of two haplotypes and underlined letter represents the mutant alleles in each position.

No.	*vgsc* genotype (989-1016-1534-1763)	Dead			Live			Survival rate (%)	*p* value
		Tainan City	Kaohsiung City	Total	Tainan City	Kaohsiung City	Total		
1	SVFD/SVFD	7	38	45	0	0	0	0	-
2	SVFD/SVCD	2	10	12	1	0	1	7.7	n.s.
3	SVFD/SGFY	4	9	13	0	0	0	0	n.s.
4	SVFD/SVFY	1	0	1	0	0	0	0	n.s.
5	SGFD/SVFD	0	1	1	0	1	1	50	0.0426
6	SVCD/SVCD	2	0	2	0	2	2	50	0.0051
7	SVFD/PGFD	2	2	4	0	4	4	50	0.0002
8	SVCD/PGFD	0	0	0	3	11	14	100	0.0000
9	SVCD/SGFY	0	0	0	1	3	4	100	0.0000
10	SGFY/PGFD	0	0	0	2	2	4	100	0.0000
11	SGFY/SGFY	0	0	0	2	2	4	100	0.0000
12	PGFD/PGFD	0	0	0	0	3	3	100	0.0001
Total		18	60	78	9	28	37	33.3	

n.s. denotes not significant.

### *vgsc* genotype and cypermethrin resistance

**Fig 4:**
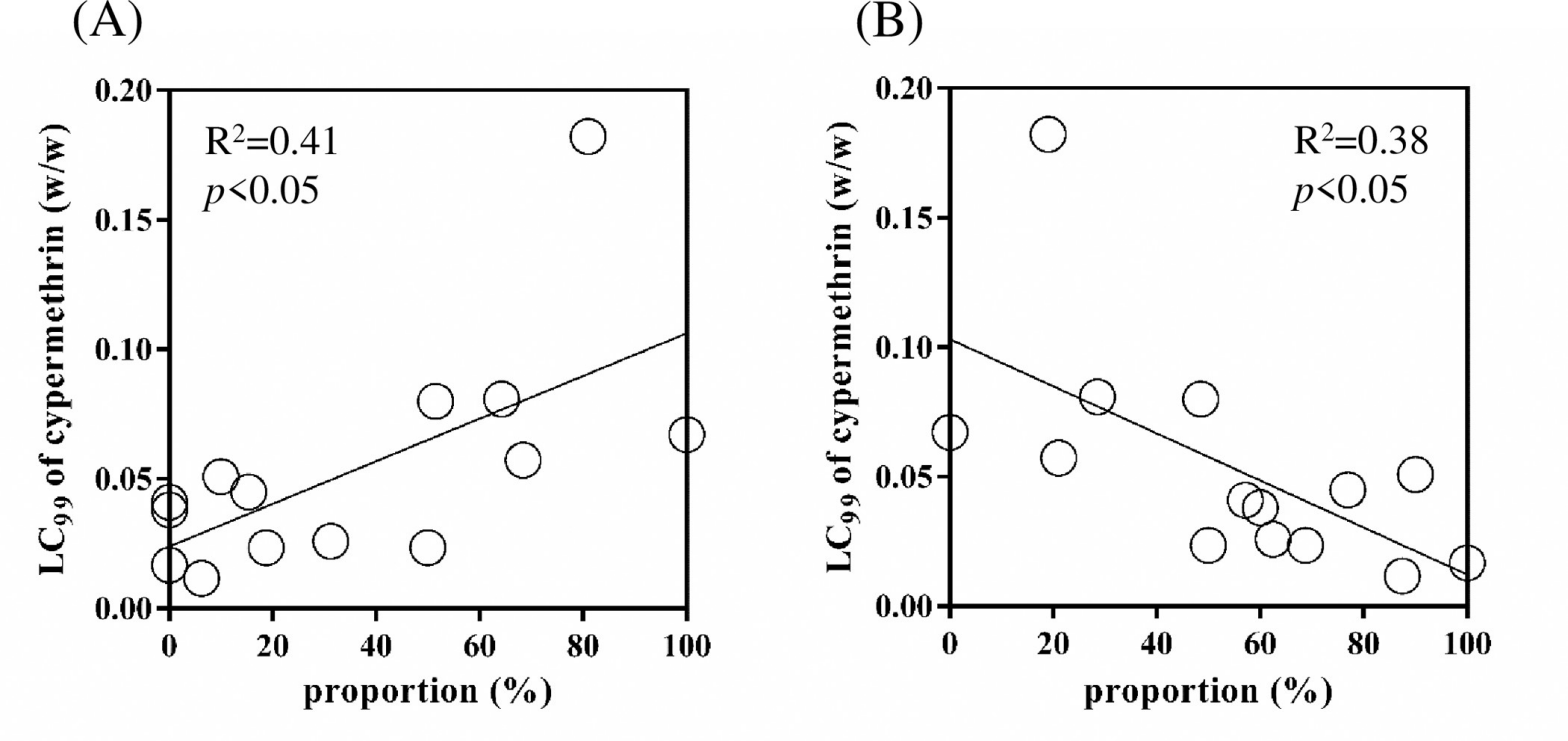
Relationship between cypermethrin susceptibility and totalized proportion of specific *vgsc* genotypes. (A) Resistance associated 7 *vgsc* genotypes or (B) Resistance unrelated 4 *vgsc* genotypes were correlated with the LC99 of cypermethrin in each district. Open circles represent administrative districts in either March or October. R^2^ was calculated by Pearson’s correlation analysis.

The proportion of *vgsc* genotypes versus LC_99_ of cypermethrin in each administrative district was analyzed by Pearson’s regression model (Fig 4). A significant positive correlation (R^2^ = 0.41, *p* < 0.05) was found between the LC_99_ of cypermethrin and the proportion of 7 resistance genotypes (SVCD/SVCD, SVFD/PGFD, SVCD/PGFD, SVCD/SGFY, SGFY/PGFD, SGFY/SGFY, and PGFD/PGFD) (Fig 4A). A significant negative correlation (R^2^ = 0.38, *p* < 0.05) between the LC_99_ of cypermethrin and the proportion of the wild type (SVFD/SVFD) and the other 3 genotypes (SVFD/SVCD, SVFD/SGFY, and SVFD/SVFY) was observed (Fig 4B).

## Discussion

In this study, mosquitoes were collected before (March) and during dengue season (October) since large scale insecticide interventions were applied in response to the dengue outbreak. We found mutant *vgsc* allele frequencies for amino acid positions 989, 1016, 1534, and 1763 in mosquitoes collected in October were significantly higher than those collected in March. The distribution of zygosity type of each allele shifted from March to October. Furthermore, in-depth analysis showed that combined genotypes for four loci significantly increased in October, implying their resistance role. Similar results in the cypermethrin exposure bioassay supported these field observations. Moreover, a positive association between the proportion of resistance genotypes and LC_99_ was observed. Here, we addressed the role of combined *vgsc* genotypes in natural populations of *Ae*. *aegypti* in Taiwan and suggested the use of combined *vgsc* genotype as an alternative method for resistance evaluation.

The association of the *vgsc* allele with resistance has been well documented [39]. In this study, the frequencies of four mutant *vgsc* alleles were significantly increased in October compared with March. Moreover, S989P and V1016G associated with cypermethrin resistance were observed in cypermethrin exposure bioassay. Our results were consistent with the following studies. In Du et al.’s study, the resistance role of V1016G was biologically confirmed in the *Xenopus* oocyte expression system. The coexistence of S989P was demonstrated to enhance the insensitivity conferred by V1016G by Hirata et al. [26,28]. Moreover, our previous study showed that the frequent concurrence of S989P and V1016G was due to the coexistence of the same haplotype and demonstrated the association between the S989P+V1016G haplotype and resistance in Taiwanese *Ae*. *aegypti* [33]. Smith et al.’s reported that S989P+V1016G mutation in *Ae*. *aegypti* confers resistance to diverse pyrethroids [40]. On the other hand, the reduction of pyrethroid sensitivity by F1534C was biologically confirmed in previous studies [26,28]. In addition, the coexistence of D1763Y and V1016G was observed in both permethrin-selected strains and field populations of *Ae*. *aegypti* in Taiwan[32,33,41]. However, in the present study, we only showed that the mutation frequency at these *vgsc* sites was significantly higher in mosquitoes collected in October but was not supported by the bioassay data (Figs 3 and 4). Although the mean mutation frequency of this allele of alive mosquitoes was higher than that of dead mosquitoes, no significance was detected. This may be due to the small sample size and outliers.

Previous studies have reported pyrethroid resistance in *Ae*. *aegypti* associated with the combined *vgsc* mutations of 989, 1011, 1016, and 1534 alleles [42–46]. Here, we detected that 6 genotypes out of the 16 genotypes were significantly increased in field *Ae*. *aegypti* populations collected in October. We also detected 8 resistance genotypes in a total of 12 genotypes after a cypermethrin bioassay in G1 females (7 resistance genotypes in a total of 13 genotypes when the result of lab strain was included). These slight differences between G0 and G1 were because the absence of SGFD/SVCD and SVFD/PVFD and small number of mosquito harboring SP/VG/FC/DY in October as well as only one mosquito with SVFD/SVFY observed in the bioassay. These resistance genotypes revealed in our field data as well as bioassay data were consistent with previous studies. These studies reported that PGF/PGF, PGF/SVC, SGFY/SGFY, SVC/SVC and PGF/SVF confer insensitivity in either the *Xenopus* oocyte expression system or crossing experiments or pyrethroid selection experiments [26,28,32,47]. In addition, we first described the other 2 resistance genotypes (SVCD/SGFY, SGFY/PGFD and the mutation genotypes that did not confer resistance.

In our previous study, four *vgsc* mutations (989, 1016, 1534, and 1763) were reported in field *Ae*. *aegypti* in Taiwan. Both S989P and V1016G are associated with resistance. We also suggested the haplotype with S989P+V1016G as a reference for resistance monitoring [33]. In this study, we detected 7 resistance genotypes in the bioassay, most of which possess S989P, V1016G or both, and these results were consistent with our previous study [33]. Moreover, we further pointed out the resistance role of the SVCD/SVCD genotype (Table 2 No. 12 and Table 4 No. 6). F1534C homozygous-dependent resistance has been described previously [26,28]. However, a significant role was not observed in our F1534C allele-specific analysis (Tables 1 and 3) or in our previous study [33]. We speculate that this may be because the F1534C homozygous mutation was believed to confer relatively mild resistance [28,47] and was confounded when a population contained other *vgsc* mutations that were more potent to resistance, such as PGFD/PGFD and SVCD/PGFD. A similar result was also reported in a previous study in which the negative role of F1534C was observed in the presence of S989P and V1016G [48].

Both the four *vgsc* alleles and specific genotypes were associated with resistance in this study. We suggest that evaluation of resistance using the combined *vgsc* genotype is preferable. In contrast to evaluating each *vgsc* mutation separately, the genotype revealed the co-occurrence of *vgsc* mutations. For example, a population composed of the genotypes SVFD/SVCD and SVFD/SGFY (or SVFD/SVCD and SVFD/PGFD) show similar alleles frequency but different resistance phenotype with a population with SVFD/ SVFD and SVCD/SGFY (or SVFD/ SVFD and SVCD/PGFD). The survival rate in SVFD/SVFD, SVFD/SVCD, SVFD/SGFY, and SVFD/PGFD were 0%, 7.7%, 0%, and 50% (Table 4 No. 2, 3, and 7), respectively. However, a synergistic effect was observed when two mutant haplotypes were combined. The survival rates of SVCD/PGFD, SVCD/SGFY, and SGFY/PGFD were all 100% (Table 4 No. 8, 9, and 10). These results also supported the previous report that the resistance role of heterozygous mutation depends on the coexistence of other mutations on the other haplotype, although *vgsc* mutations are generally believed to be recessive [47,49,50]. Moreover, our results also showed that combined *vgsc* genotype are more accurate than haplotype for resistance prediction. Genotype clearly describes the status of homozygous/heterozygous mutations that play an important role in resistance. For instance, double homozygous of V1016G+D1763Y and S989P+V1016G significantly protect mosquitoes against cypermethrin than those of mosquitoes with heterozygous mutations. A similar observation was also found in F1534C (Table 4 No. 2 v.s. No. 6, No. 3 v.s. No. 11 and No. 7 v.s. No. 12). According to these results, we proposed that a resistance estimation would be more accurate when assessing by the genotype combination rather than haplotype or each allele, especially when the mutations occur in the different haplotypes [33]. Furthermore, we detected that several *vgsc* genotypes did not confer resistance; these *vgsc* genotypes contributing to allele frequency will not provide significant resistance and should be taken into consideration when accessing resistance (Table 4 No. 2, 3, 4). For these reasons, we emphasize that the combined *vgsc* genotype is an accurate tool for resistance prediction.

In this study, we specified the role of *vgsc* genotypes (S989P, V1016G, F1534C and D1763Y) in insecticide resistance in Taiwan. Until now, the distribution of *vgsc* mutations are geographically limited. Different resistance roles of *Ae*. *aegypti* genotypes with different alleles and haplotypes in Asia, America and Africa are well documented [51]. The V1016I, I1011M and F1534C mutations occurred in America and the V1016I and F1534C mutations in Africa. Furthermore, globalization and international trade provide the opportunity that two distinct lines of mosquitoes with different *vgsc* mutations might interact and produce new *vgsc* genotypes [52]. We urge that authorities should monitor the invasion of mosquitoes in international airports and ports with genetic resistance surveillance.

Application of insecticides is still one of the important measures for prevention of arbovirus transmission. Therefore, insecticide resistance monitoring is a prerequisite element for effective interventions. Early and accurate detection of resistance is crucial for adjusting the vector control program to limit the exacerbation of resistance and minimize its impact on the efforts of disease prevention and control. However, traditional diagnostic bioassays requires insectary facilities for rearing large quantity of female mosquitoes which is time-consuming. It usually takes at least 1–2 months and these delayed results cannot reflect the real-time resistance status in the field. Molecular methods using resistance-associated alleles and haplotypes for monitoring resistance of disease vectors were proposed to compensate for the weakness of bioassay. Allele genotyping has been used in our routine insecticide resistance monitoring since 2016. We collected the field mosquitoes in March and October due to the reason stated as followings. In Taiwan, dengue is not an indigenous disease but a travelling disease, which the dengue virus is introduced in summer, transported to local mosquitoes and causes an outbreak. In general, the outbreak finish during the winter. At least 100 mosquitoes (10 males per district in the total of 10 districts in 3 cities) per season were sequenced and genotype analysis conducted. These allele frequencies along with the data of the previous years were provided to our dengue control division for reference on implementing dengue vector control program. Our study showed the combined *vgsc* genotype was more accurate than allele and haplotype to predict the resistance phenotype in field mosquitoes. Since the combined genotype was detected by traditional PCR and sequencing based on haplotype, which have been established in many *Ae*. *aegypti* populations [22], it would not cause much burden on analysis. Therefore, we suggest to include combined *vgsc* genotype/resistance phenotype profile in local mosquito population in insecticide resistance monitoring program in the area that need use cypermethrin to control *Ae*. *aegypti*.

In conclusion, the role of *vgsc* genotype composed of four mutations (989, 1016, 1534, and 1763) was determined in this study. As the usage of insecticides is still the major strategy to combat mosquito-borne diseases during outbreaks, early and accurate detection of insecticide resistance is crucial for the effectiveness of vector control. Here, we presented a method to detect mosquito resistance and evaluate the resistance level by resistance-associated combined *vgsc* genotypes, which can provide real-time information for decision makers regarding the adoption of appropriate vector control strategies.

## Supporting information

S1 Table The PCR and sequence primers used in this study. (DOCX)

S2 Table *vgsc* mutation frequencies of *Ae*. *aegypti* in each district of Tainan city, Kaohsiung city and Pingtung city in March and October. Wild type, heterozygous mutation, homozygous mutation and allele frequency are abbreviated as SS, SR, RR and AF
(DOCX)

S3 Table The distribution of *vgsc* genotypes (for single amino acid sites) in live and dead female mosquitos after cypermethrin exposure bioassay. Underlined letter represents the mutant alleles in each position.
(DOCX)

S4 Table The distribution of combined *vgsc* genotypes in live and dead female after cypermethrin exposure bioassay. Genotype was described as the composition of two haplotypes and underlined letter represents the mutant alleles in each position. Tainan city, Kaohsiung city and Lab strain are abbreviated to TN, KH and LS.
(DOCX)

S5 Table *vgsc* genotypes of live and dead mosquitoes after cypermethrin exposure. Boxes with white, gray and yellow represent wild type, heterozygous mutation and homozygous mutation, respectively. The 3 codons listed in the box present the nucleotide sequences of the amino acid in the site of 989, 1016, 1534, and 1763 detected by Sanger sequencing.
(DOCX)
