# Supplementary material for: The role of voltage-gated sodium channel genotypes in pyrethroid resistance in Aedes aegypti in Taiwan
Source: PLoS Negl Trop Dis. 2022 Sep 22;16(9):e0010780. doi: 10.1371/journal.pntd.0010780 (PMC9531798; doi:10.1371/journal.pntd.0010780)
Supplement: S1 Table — (DOCX) [file pntd.0010780.s001.docx]

**S1 Table.** **The PCR and sequence primers used in this study.**

| Primer name | Sequence | Experiment | Target site |
| --- | --- | --- | --- |
| AaSCF20 | GACAATGTGGATCGCTTCCC | PCR | domain II (Forward) |
| AaSCR21 | GCAATCTGGCTTGTTAACTTG |  | domain II (Reverse) |
| AaSCF7 | GAGAACTCGCCGATGAACTT |  | domain III (Forward) |
| AaSCR7 | GACGACGAAATCGAACAGGT |  | domain III (Reverse) |
| AlSCF6 | TCGAGAAGTACTTCGTGTCG |  | domain IV (Forward) |
| AlSCR8 | AACAGCAGGATCATGCTCTG |  | domain IV (Reverse) |
| AaSCF3* | GTGGAACTTCACCGACTTCA | Sequencing | domain II (Forward) |
| AaSCR22* | TTCACGAACTTGAGCGCGTTG |  | domain II (Reverse) |
| AaSCR8 | TAGCTTTCAGCGGCTTCTTC |  | domain III (Reverse) |
| AlSCF7 | AGGTATCCGAACGTTGCTGT |  | domain IV (Forward) |

*Both the AaSCF3 and AaSCR22 primers were used for domain II sequencing in case of heterozygous intron polymorphisms existed between exon 20 and 21.
