# Supplementary material for: The role of voltage-gated sodium channel genotypes in pyrethroid resistance in Aedes aegypti in Taiwan
Source: PLoS Negl Trop Dis. 2022 Sep 22;16(9):e0010780. doi: 10.1371/journal.pntd.0010780 (PMC9531798; doi:10.1371/journal.pntd.0010780)
Supplement: S2 Table — Wild type, heterozygous mutation, homozygous mutation and allele frequency are abbreviated as SS, SR, RR and AF (DOCX) [file pntd.0010780.s002.docx]

**S2 Table.** ***vgsc* mutation frequencies of *Ae. aegypti* in each district of Tainan city, Kaohsiung city and Pingtung city in March and October**. Wild type, heterozygous mutation, homozygous mutation and allele frequency are abbreviated as SS, SR, RR and AF

| Month | District | No. | S989P | | | | V1016G | | | | F1534C | | | | D1763Y | | | |
| --- | --- | --- | --- | --- | --- | --- | --- | --- | --- | --- | --- | --- | --- | --- | --- | --- | --- | --- |
|  |  |  | SS | SR | RR | AF | SS | SR | RR | AF | SS | SR | RR | AF | SS | SR | RR | AF |
| March | West Central | 10 | 10 | 0 | 0 | 0 | 6 | 4 | 0 | 0.2 | 7 | 3 | 0 | 0.15 | 10 | 0 | 0 | 0 |
|  | North | 22 | 19 | 3 | 0 | 0.07 | 16 | 6 | 0 | 0.14 | 13 | 9 | 0 | 0.20 | 20 | 2 | 0 | 0.05 |
|  | South | 12 | 10 | 1 | 1 | 0.13 | 8 | 3 | 1 | 0.21 | 10 | 2 | 0 | 0.08 | 10 | 2 | 0 | 0.08 |
|  | Yongkang | 16 | 15 | 1 | 0 | 0.03 | 13 | 3 | 0 | 0.09 | 13 | 3 | 0 | 0.09 | 15 | 1 | 0 | 0.03 |
|  | Xiaogang | 10 | 10 | 0 | 0 | 0 | 10 | 0 | 0 | 0 | 8 | 1 | 1 | 0.15 | 10 | 0 | 0 | 0 |
|  | Fengshan | 33 | 21 | 7 | 5 | 0.26 | 20 | 8 | 5 | 0.27 | 23 | 5 | 5 | 0.23 | 32 | 1 | 0 | 0.02 |
|  | Sanmin | 7 | 7 | 0 | 0 | 0 | 4 | 3 | 0 | 0.15 | 4 | 3 | 0 | 0.15 | 7 | 0 | 0 | 0 |
|  | Qianzhen | 13 | 13 | 0 | 0 | 0 | 10 | 3 | 0 | 0.12 | 7 | 4 | 2 | 0.31 | 11 | 2 | 0 | 0.08 |
|  | Pingtung | 25 | 22 | 3 | 0 | 0.06 | 21 | 4 | 0 | 0.08 | 18 | 6 | 1 | 0.16 | 24 | 1 | 0 | 0.02 |
|  | Total | 148 | 127 | 15 | 6 | 0.09 | 108 | 34 | 6 | 0.16 | 103 | 36 | 9 | 0.18 | 139 | 9 | 0 | 0.03 |
| October | West Central | 10 | 9 | 1 | 0 | 0.05 | 6 | 3 | 1 | 0.25 | 5 | 4 | 1 | 0.3 | 7 | 2 | 1 | 0.2 |
|  | North | 22 | 20 | 2 | 0 | 0.05 | 19 | 3 | 0 | 0.07 | 14 | 4 | 4 | 0.27 | 21 | 1 | 0 | 0.02 |
|  | South | 16 | 14 | 2 | 0 | 0.06 | 8 | 6 | 2 | 0.31 | 10 | 6 | 0 | 0.19 | 9 | 7 | 0 | 0.22 |
|  | Yongkang | 16 | 15 | 1 | 0 | 0.03 | 12 | 4 | 0 | 0.13 | 11 | 3 | 2 | 0.22 | 15 | 1 | 0 | 0.03 |
|  | Xiaogang | 19 | 3 | 8 | 8 | 0.63 | 2 | 7 | 10 | 0.71 | 9 | 8 | 2 | 0.32 | 17 | 2 | 0 | 0.05 |
|  | Fengshan | 28 | 14 | 6 | 8 | 0.39 | 11 | 7 | 10 | 0.48 | 18 | 7 | 3 | 0.23 | 22 | 5 | 1 | 0.13 |
|  | Sanmin | 19 | 8 | 9 | 2 | 0.34 | 4 | 11 | 4 | 0.50 | 8 | 10 | 1 | 0.32 | 14 | 4 | 1 | 0.16 |
|  | Qianzhen | 21 | 8 | 7 | 6 | 0.45 | 6 | 7 | 8 | 0.55 | 12 | 7 | 2 | 0.26 | 17 | 4 | 0 | 0.10 |
|  | Pingtung | 21 | 9 | 9 | 3 | 0.36 | 5 | 11 | 5 | 0.50 | 8 | 11 | 2 | 0.36 | 16 | 4 | 1 | 0.14 |
|  | Total | 172 | 100 | 45 | 27 | 0.29 | 73 | 59 | 40 | 0.40 | 95 | 60 | 17 | 0.27 | 138 | 30 | 4 | 0.11 |
| Total | | 320 | 227 | 60 | 33 | 0.20 | 181 | 93 | 46 | 0.29 | 198 | 96 | 26 | 0.23 | 277 | 39 | 4 | 0.07 |
