# Supplementary material for: The role of voltage-gated sodium channel genotypes in pyrethroid resistance in Aedes aegypti in Taiwan
Source: PLoS Negl Trop Dis. 2022 Sep 22;16(9):e0010780. doi: 10.1371/journal.pntd.0010780 (PMC9531798; doi:10.1371/journal.pntd.0010780)
Supplement: S3 Table — Underlined letter represents the mutant alleles in each position. (DOCX) [file pntd.0010780.s003.docx]

**S3 Table. The distribution of *vgsc* genotypes (for single amino acid sites) in live and dead female mosquitos after cypermethrin exposure bioassay***.* Underlined letter represents the mutant alleles in each position

| *vgsc*  genotypes | | Tainan city | | Kaohsiung city | | Lab strain | | Total | | p value |
| --- | --- | --- | --- | --- | --- | --- | --- | --- | --- | --- |
|  |  | Dead | Live | Dead | Live | Dead | Live | Dead | Live |  |
| 989 | S/S | 16 | 4 | 58 | 8 | 34 | 3 | 108 | 15 | - |
|  | S/P | 2 | 5 | 2 | 17 | 0 | 7 | 4 | 29 | 0.0000 |
|  | P/P | 0 | 0 | 0 | 3 | 0 | 2 | 0 | 5 | 0.0001 |
| 1016 | V/V | 12 | 1 | 48 | 2 | 29 | 2 | 89 | 5 | - |
|  | V/G | 6 | 4 | 12 | 19 | 5 | 5 | 23 | 28 | 0.0000 |
|  | G/G | 0 | 4 | 0 | 7 | 0 | 5 | 0 | 16 | 0.0000 |
| 1534 | F/F | 14 | 4 | 50 | 12 | 24 | 6 | 88 | 22 | - |
|  | F/C | 2 | 5 | 10 | 14 | 10 | 5 | 22 | 24 | 0.0001 |
|  | C/C | 2 | 0 | 0 | 2 | 0 | 1 | 2 | 3 | 0.0600 |
| 1763 | D/D | 13 | 4 | 51 | 21 | 12 | 9 | 76 | 34 | - |
|  | D/Y | 5 | 3 | 9 | 5 | 22 | 2 | 36 | 10 | 0.0817 |
|  | Y/Y | 0 | 2 | 0 | 2 | 0 | 1 | 0 | 5 | 0.0038 |
