# Supplementary material for: The role of voltage-gated sodium channel genotypes in pyrethroid resistance in Aedes aegypti in Taiwan
Source: PLoS Negl Trop Dis. 2022 Sep 22;16(9):e0010780. doi: 10.1371/journal.pntd.0010780 (PMC9531798; doi:10.1371/journal.pntd.0010780)
Supplement: S4 Table — Genotype was described as the composition of two haplotypes and underlined letter represents the mutant alleles in each position. Tainan city, Kaohsiung city and Lab strain are abbreviated to TN, KH and LS. (DOCX) [file pntd.0010780.s004.docx]

**S4 Table. The distribution of combined *vgsc* genotypes in live and dead female after cypermethrin exposure bioassay**. Genotype was described as the composition of two haplotypes and underlined letter represents the mutant alleles in each position. Tainan city, Kaohsiung city and Lab strain are abbreviated to TN, KH and LS

| No. | *vgsc* genotype  (989-1016-1534-1763) | Dead | | | | Live | | | | Survival  rate (%) | p value |
| --- | --- | --- | --- | --- | --- | --- | --- | --- | --- | --- | --- |
|  |  | TN | KH | LS | Total | TN | KH | LS | Total |  |  |
| 1 | SVFD/SVFD | 7 | 38 | 8 | 53 | 0 | 0 | 1 | 1 | 1.8 | - |
| 2 | SVFD/SVCD | 2 | 10 | 4 | 16 | 1 | 0 | 0 | 1 | 5.9 | n.s |
| 3 | SVFD/SGFY | 4 | 9 | 5 | 18 | 0 | 0 | 0 | 0 | 0 | n.s |
| 4 | SVFD/SVFY | 1 | 0 | 11 | 12 | 0 | 0 | 0 | 0 | 0 | n.s |
| 5 | SS/VV/FC/DY* | 0 | 0 | 6 | 6 | 0 | 0 | 0 | 0 | 0 | n.s |
| 6 | SGFD/SVFD | 0 | 1 | 0 | 1 | 0 | 1 | 0 | 1 | 50 | n.s |
| 7 | SVCD/SVCD | 2 | 0 | 0 | 2 | 0 | 2 | 1 | 3 | 60 | 0.0012 |
| 8 | SVFD/PGFD | 2 | 2 | 0 | 4 | 0 | 4 | 0 | 4 | 50 | 0.0006 |
| 9 | SVCD/PGFD | 0 | 0 | 0 | 0 | 3 | 11 | 5 | 19 | 100 | 0.0000 |
| 10 | SVCD/SGFY | 0 | 0 | 0 | 0 | 1 | 3 | 0 | 4 | 100 | 0.0000 |
| 11 | SGFY/PGFD | 0 | 0 | 0 | 0 | 2 | 2 | 2 | 6 | 100 | 0.0000 |
| 12 | SGFY/SGFY | 0 | 0 | 0 | 0 | 2 | 2 | 1 | 5 | 100 | 0.0000 |
| 13 | PGFD/PGFD | 0 | 0 | 0 | 0 | 0 | 3 | 2 | 5 | 100 | 0.0000 |
| Total | | 18 | 60 | 34 | 112 | 9 | 28 | 12 | 49 | 30.4 |  |

* Haplotypes cannot be proposed according to previous study [1]

Reference

1. Chung HH, Cheng IC, Chen YC, Lin C, Tomita T, Teng HJ. Voltage-gated sodium channel intron polymorphism and four mutations comprise six haplotypes in an Aedes aegypti population in Taiwan. PLoS Negl Trop Dis. 2019;13(3):e0007291. Epub 2019/03/30. doi: 10.1371/journal.pntd.0007291. PubMed PMID: 30925149; PubMed Central PMCID: PMCPMC6457567.
