# Supplementary material for: The role of voltage-gated sodium channel genotypes in pyrethroid resistance in Aedes aegypti in Taiwan
Source: PLoS Negl Trop Dis. 2022 Sep 22;16(9):e0010780. doi: 10.1371/journal.pntd.0010780 (PMC9531798; doi:10.1371/journal.pntd.0010780)
Supplement: S5 Table — Boxes with white, gray and yellow represent wild type, heterozygous mutation and homozygous mutation, respectively. The 3 codons listed in the box present the nucleotide sequences of the amino acid in the site of 989, 1016, 1534, and 1763 detected by Sanger sequencing. (DOCX) [file pntd.0010780.s005.docx]

**S5 Table. *vgsc* genotypes of live and dead mosquitoes after cypermethrin exposure.** Boxes with white, gray and yellow represent wild type, heterozygous mutation and homozygous mutation, respectively. The 3 codons listed in the box present the nucleotide sequences of the amino acid in the site of 989, 1016, 1534, and 1763 detected by Sanger sequencing

| **District** | **Phenotype** | ***vgsc* genotype** | | | |
| --- | --- | --- | --- | --- | --- |
|  |  | **989** | **1016** | **1534** | **1763** |
| Qianzhen | live | CCC homo | GGA homo | wild type | wild type |
| Qianzhen | live | CCC homo | GGA homo | wild type | wild type |
| Xiaogang | live | CCC homo | GGA homo | wild type | wild type |
| Qianzhen | live | TCC/CCC hetero | GGA homo | wild type | GAC/TAC hetero |
| Qianzhen | live | TCC/CCC hetero | GGA homo | wild type | GAC/TAC hetero |
| West Central | live | TCC/CCC hetero | GGA homo | wild type | GAC/TAC hetero |
| South | live | TCC/CCC hetero | GGA homo | wild type | GAC/TAC hetero |
| Qianzhen | live | TCC/CCC hetero | GTA/GGA hetero | GAA/GCA hetero | wild type |
| Qianzhen | live | TCC/CCC hetero | GTA/GGA hetero | GAA/GCA hetero | wild type |
| Qianzhen | live | TCC/CCC hetero | GTA/GGA hetero | GAA/GCA hetero | wild type |
| Qianzhen | live | TCC/CCC hetero | GTA/GGA hetero | GAA/GCA hetero | wild type |
| Qianzhen | live | TCC/CCC hetero | GTA/GGA hetero | GAA/GCA hetero | wild type |
| Qianzhen | live | TCC/CCC hetero | GTA/GGA hetero | GAA/GCA hetero | wild type |
| Qianzhen | live | TCC/CCC hetero | GTA/GGA hetero | GAA/GCA hetero | wild type |
| Qianzhen | live | TCC/CCC hetero | GTA/GGA hetero | GAA/GCA hetero | wild type |
| Fengshan | live | TCC/CCC hetero | GTA/GGA hetero | GAA/GCA hetero | wild type |
| South | live | TCC/CCC hetero | GTA/GGA hetero | GAA/GCA hetero | wild type |
| South | live | TCC/CCC hetero | GTA/GGA hetero | GAA/GCA hetero | wild type |
| South | live | TCC/CCC hetero | GTA/GGA hetero | GAA/GCA hetero | wild type |
| Xiaogang | live | TCC/CCC hetero | GTA/GGA hetero | GAA/GCA hetero | wild type |
| Xiaogang | live | TCC/CCC hetero | GTA/GGA hetero | GAA/GCA hetero | wild type |
| Qianzhen | live | TCC/CCC hetero | GTA/GGA hetero | wild type | wild type |
| Qianzhen | live | TCC/CCC hetero | GTA/GGA hetero | wild type | wild type |
| Qianzhen | live | TCC/CCC hetero | GTA/GGA hetero | wild type | wild type |
| Qianzhen | live | TCC/CCC hetero | GTA/GGA hetero | wild type | wild type |
| Fengshan | live | wild type | GGA homo | wild type | TAC homo |
| South | live | wild type | GGA homo | wild type | TAC homo |
| South | live | wild type | GGA homo | wild type | TAC homo |
| Xiaogang | live | wild type | GGA homo | wild type | TAC homo |
| Qianzhen | live | wild type | GTA/GGA hetero | GAA/GCA hetero | GAC/TAC hetero |
| Qianzhen | live | wild type | GTA/GGA hetero | GAA/GCA hetero | GAC/TAC hetero |
| West Central | live | wild type | GTA/GGA hetero | GAA/GCA hetero | GAC/TAC hetero |
| Fengshan | live | wild type | GTA/GGA hetero | GAA/GCA hetero | GAC/TAC hetero |
| Sanmin | live | wild type | GTA/GGA hetero | wild type | wild type |
| South | live | wild type | wild type | GAA/GCA hetero | wild type |
| Qianzhen | live | wild type | wild type | GCA homo | wild type |
| Qianzhen | live | wild type | wild type | GCA homo | wild type |
| Qianzhen | dead | TCC/CCC hetero | GTA/GGA hetero | wild type | wild type |
| Qianzhen | dead | TCC/CCC hetero | GTA/GGA hetero | wild type | wild type |
| South | dead | TCC/CCC hetero | GTA/GGA hetero | wild type | wild type |
| South | dead | TCC/CCC hetero | GTA/GGA hetero | wild type | wild type |
| Qianzhen | dead | wild type | GTA/GGA hetero | wild type | GAC/TAC hetero |
| Qianzhen | dead | wild type | GTA/GGA hetero | wild type | GAC/TAC hetero |
| Qianzhen | dead | wild type | GTA/GGA hetero | wild type | GAC/TAC hetero |
| Qianzhen | dead | wild type | GTA/GGA hetero | wild type | GAC/TAC hetero |
| West Central | dead | wild type | GTA/GGA hetero | wild type | GAC/TAC hetero |
| Fengshan | dead | wild type | GTA/GGA hetero | wild type | GAC/TAC hetero |
| Fengshan | dead | wild type | GTA/GGA hetero | wild type | GAC/TAC hetero |
| Fengshan | dead | wild type | GTA/GGA hetero | wild type | GAC/TAC hetero |
| South | dead | wild type | GTA/GGA hetero | wild type | GAC/TAC hetero |
| South | dead | wild type | GTA/GGA hetero | wild type | GAC/TAC hetero |
| South | dead | wild type | GTA/GGA hetero | wild type | GAC/TAC hetero |
| Xiaogang | dead | wild type | GTA/GGA hetero | wild type | GAC/TAC hetero |
| Xiaogang | dead | wild type | GTA/GGA hetero | wild type | GAC/TAC hetero |
| Qianzhen | dead | wild type | GTA/GGA hetero | wild type | wild type |
| Qianzhen | dead | wild type | wild type | GAA/GCA hetero | wild type |
| Qianzhen | dead | wild type | wild type | GAA/GCA hetero | wild type |
| Qianzhen | dead | wild type | wild type | GAA/GCA hetero | wild type |
| Qianzhen | dead | wild type | wild type | GAA/GCA hetero | wild type |
| Qianzhen | dead | wild type | wild type | GAA/GCA hetero | wild type |
| Qianzhen | dead | wild type | wild type | GAA/GCA hetero | wild type |
| West Central | dead | wild type | wild type | GAA/GCA hetero | wild type |
| West Central | dead | wild type | wild type | GAA/GCA hetero | wild type |
| Fengshan | dead | wild type | wild type | GAA/GCA hetero | wild type |
| Fengshan | dead | wild type | wild type | GAA/GCA hetero | wild type |
| Fengshan | dead | wild type | wild type | GAA/GCA hetero | wild type |
| Xiaogang | dead | wild type | wild type | GAA/GCA hetero | wild type |
| West Central | dead | wild type | wild type | GCA homo | wild type |
| South | dead | wild type | wild type | GCA homo | wild type |
| South | dead | wild type | wild type | wild type | GAC/TAC hetero |
| Qianzhen | dead | wild type | wild type | wild type | wild type |
| Qianzhen | dead | wild type | wild type | wild type | wild type |
| Qianzhen | dead | wild type | wild type | wild type | wild type |
| Qianzhen | dead | wild type | wild type | wild type | wild type |
| Qianzhen | dead | wild type | wild type | wild type | wild type |
| Qianzhen | dead | wild type | wild type | wild type | wild type |
| Qianzhen | dead | wild type | wild type | wild type | wild type |
| Qianzhen | dead | wild type | wild type | wild type | wild type |
| Qianzhen | dead | wild type | wild type | wild type | wild type |
| Qianzhen | dead | wild type | wild type | wild type | wild type |
| Qianzhen | dead | wild type | wild type | wild type | wild type |
| Qianzhen | dead | wild type | wild type | wild type | wild type |
| Qianzhen | dead | wild type | wild type | wild type | wild type |
| Qianzhen | dead | wild type | wild type | wild type | wild type |
| Qianzhen | dead | wild type | wild type | wild type | wild type |
| Qianzhen | dead | wild type | wild type | wild type | wild type |
| Qianzhen | dead | wild type | wild type | wild type | wild type |
| Qianzhen | dead | wild type | wild type | wild type | wild type |
| Qianzhen | dead | wild type | wild type | wild type | wild type |
| Qianzhen | dead | wild type | wild type | wild type | wild type |
| West Central | dead | wild type | wild type | wild type | wild type |
| Fengshan | dead | wild type | wild type | wild type | wild type |
| Fengshan | dead | wild type | wild type | wild type | wild type |
| Fengshan | dead | wild type | wild type | wild type | wild type |
| Fengshan | dead | wild type | wild type | wild type | wild type |
| Fengshan | dead | wild type | wild type | wild type | wild type |
| South | dead | wild type | wild type | wild type | wild type |
| South | dead | wild type | wild type | wild type | wild type |
| South | dead | wild type | wild type | wild type | wild type |
| South | dead | wild type | wild type | wild type | wild type |
| South | dead | wild type | wild type | wild type | wild type |
| South | dead | wild type | wild type | wild type | wild type |
| Xiaogang | dead | wild type | wild type | wild type | wild type |
| Xiaogang | dead | wild type | wild type | wild type | wild type |
| Xiaogang | dead | wild type | wild type | wild type | wild type |
| Xiaogang | dead | wild type | wild type | wild type | wild type |
| Xiaogang | dead | wild type | wild type | wild type | wild type |
| Xiaogang | dead | wild type | wild type | wild type | wild type |
| Xiaogang | dead | wild type | wild type | wild type | wild type |
| Xiaogang | dead | wild type | wild type | wild type | wild type |
| Xiaogang | dead | wild type | wild type | wild type | wild type |
| Xiaogang | dead | wild type | wild type | wild type | wild type |
| Xiaogang | dead | wild type | wild type | wild type | wild type |
| Xiaogang | dead | wild type | wild type | wild type | wild type |
| Xiaogang | dead | wild type | wild type | wild type | wild type |
